# Comparison of Different 2D and 3D-QSAR Methods on Activity Prediction of Histamine H3 Receptor Antagonists

**Published:** 2012

**Authors:** Siavoush Dastmalchi, Maryam Hamzeh-Mivehroud, Karim Asadpour-Zeynali

**Affiliations:** a *DepartmentofMedicinalChemistry, SchoolofPharmacy, TabrizUniversityofMedicalSciences, Tabriz, Iran. *; b *BiotechnologyResearchCenter, TabrizUniversityofMedicalSciences, Tabriz, Iran. *; c *DepartmentofAnalyticalChemistry, FacultyofChemistry, UniversityofTabriz, Tabriz, Iran.*

**Keywords:** Histamine H3 receptor, QSAR, HASL, Multiple linear regression, Neural network.

## Abstract

Histamine H3 receptor subtype has been the target of several recent drug development programs. Quantitative structure-activity relationship (QSAR) methods are used to predict the pharmaceutically relevant properties of drug candidates whenever it is applicable. The aim of this study was to compare the predictive powers of three different QSAR techniques, namely, multiple linear regression (MLR), artificial neural network (ANN), and HASL as a 3D QSAR method, in predicting the receptor binding affinities of arylbenzofuran histamine H3 receptor antagonists. Genetic algorithm coupled partial least square as well as stepwise multiple regression methods were used to select a number of calculated molecular descriptors to be used in MLR and ANN-based QSAR studies. Using the leave-group-out cross-validation technique, the performances of the MLR and ANN methods were evaluated. The calculated values for the mean absolute percentage error (MAPE), ranging from 2.9 to 3.6, and standard deviation of error of prediction (SDEP), ranging from 0.31 to 0.36, for both MLR and ANN methods were statistically comparable, indicating that both methods perform equally well in predicting the binding affinities of the studied compounds toward the H3 receptors. On the other hand, the results from 3D-QSAR studies using HASL method were not as good as those obtained by 2D methods. It can be concluded that simple traditional approaches such as MLR method can be as reliable as those of more advanced and sophisticated methods like ANN and 3D-QSAR analyses.

## Introduction

Histamine is a hydrophilic biological amine which is widely distributed throughout the animal kingdom. Almost all mammalian tissues contain histamine in varying amounts and consistent with its wide tissue distribution, it involves in many important physiological functions such as allergic responses and regulation of gastric acid secretion (1). In the peripheral and central nervous systems, it functions as a neurotransmitter (2). The wide range of physiological effects of histamine resultfrom its recognition through specific cell-surface receptors belonging to the G-protein coupled receptors superfamily. Pharmacological investigations suggest the existence of multiple receptor subtypes for histamine. Up to now, four different receptors were cloned and designated histamine H1 to H4 receptor subtypes (3-6). Histamine H3 receptor identified in 1983, regulates the synthesis and release of histamine through a negative feedback mechanism (7). Histamine H3 receptors also modulate the release of several neurotransmitters such as glutamate, acetylcholine, noradrenaline, dopamine, GABA and serotonin. It has been suggested that the H3 receptor antagonists may play a role in the treatment of several neurological diseases such as epilepsy, obesity, arousal, attention-deficit hyperactivity disorder (ADHD), schizophrenia, Alzheimer’s and Parkinson’s diseases. Thus, finding the potent and efficacious H3 receptor antagonists have been the focus of several recent drug development programs (8-12).

Computer-aided drug discovery techniques have tremendous effect in shortening the process of drug discovery investigations (13, 14). Among different computational techniques, the quantitative structure-activity relationship (QSAR) methods are certainly the major factors in the contemporary drug design. Thus, it is quite clear why the industrial units are the prime users of the QSAR methods (15). Different two- and three-dimensional QSAR techniques, such as methods based on multiple linear regression (MLR), principal component analysis (PCA), artificial neural networks (ANN) (16), and 3D GRID-based methods, like hypothetical active site lattice (HASL) and comparative molecular field analysis (CoMFA), are used to quantitatively predict the desired properties. ANN is a learning system based on a computational technique, which attempts to simulate the neurological processing ability of the brain (17). Recently, evolutionary methods such as genetic algorithm (GA) have received increasing attention for variable selection. The 3D-QSAR methods apply empirical force field calculations on the three-dimensionally aligned ligand structures. The alignments are guided mostly based on the exploration of crystallographically solved ligand-receptor complexes or direct superpositioning of the ligands. CoMFA and HASL techniques are among many different available 3D-QSAR methods. CoMFA uses both interactive graphics and statistical techniques to correlate the shapes and properties of molecules with their biological activity (18, 19). HASL technique creates a QSAR model from a composite lattice generated from a series of regular orthogonal 3D grids established for each molecule (20, 21).

In the present work, different QSAR approaches, *i.e*., MLR, ANN and HASL were used to model the receptor binding affinities of the 58 arylbenzofuran derived H3 receptor antagonists and then the predictive power of the methods were compared.

## Experimental

*Biological data*

Fifty-eight arylbenzofuran derivatives with histamine H3 antagonistic activities were used in QSAR analyses (Table 1). Their binding affinities to rat and human H3 receptors are shown in Tables 2 and 3 (22).

*Molecular descriptors*

Molecular descriptors were calculated as previously described (23). Briefly, the Hyperchem software (ver. 7.0) was used to generate 3D molecular structures and energy minimize them using MM+ force field (24). Then, the structures were fully optimized based on the semiemperical method, using AM1 level of theory (25). Hyperchem, Dragon (version 3.0) and ACDlabs suite of programs (ver. 6.00) were employed to calculate the molecular descriptors. *HOMO* and *LUMO* energies, molar refractivity, hydration energy, Log P, dipole moment, surface area and total energy were calculated using Hyperchem. From 1481 different 1D, 2D and 3D molecular descriptors calculated by Dragon software those descriptors having less than 0.95 correlation were retained for further analyses (26).

Other descriptors such as Log D at different pH values, pKa, molar volume, parachor, density, surface tension and Hansch substituent hydrophobicity constant (π) were computed using ACDlabs software.

*Descriptor selection*

In order to select the minimum number of molecular descriptors to be used in the modeling steps, the genetic algorithm coupled partial least square (GA-PLS) method of Riccardo Leardi was used in MATLAB environment (ver.7.0) with the following setup: population size, 30; probability of mutation, 0.01; probability of cross over, 0.5; number of runs, 100. As a result, about 10% of many descriptors (>1000) calculated by DRAGON, Hyperchem and ACDlabs suite of programs were selected (27, 28).

**Table 1:** Chemical structures and molecular parameters of the arylbenzofuran derivatives used in the study.

**Compound**	**Benzofuran substituent**	**Phenyl substituent**	**E_HOMO**	**LogD (pH=7.4)**	**Mor19v**	**Mor30m**	**Mor18u**	**MAXDP**	**PSA**
									
p1	H	4'-CN	-8.901	2.15	1.178	0.225	-1.277	3.99	40.17
p2	H	3'-CN	-8.876	2.91	1.022	0.224	-1.249	5.19	52.53
p3	H	4'-F	-8.730	2.76	0.972	0.162	-1.561	4.73	25.61
p4	H	3'-F	-8.798	2.85	1.001	0.217	-1.153	2.55	36.61
p5	H	4'-Cl	-8.725	3.40	0.965	0.151	-1.441	4.29	16.38
p6	H	3'-Cl	-8.783	3.39	0.897	0.208	-1.587	2.56	16.38
p7	H	2'-Cl	-8.711	3.31	0.871	0.223	-1.485	5.82	16.38
p8	H	4'-CF3	-8.939	3.80	1.017	0.326	-1.385	4.46	16.38
p9	H	3'-CF3	-8.894	3.80	1.077	0.292	-1.252	2.54	16.38
p10	H	4'-Me	-8.578	3.28	1.028	0.114	-1.700	3.08	16.38
p11	H	3'-Me	-8.630	3.28	1.095	0.123	-1.240	2.55	36.61
p12	H	2'-Me	-8.674	3.28	1.066	0.201	-1.470	2.60	33.45
p13	H	4'-OCF3	-8.855	3.68	0.919	0.422	-1.371	4.18	16.38
p14	H	3'-OCF3	-8.852	3.63	0.981	0.519	-1.291	2.54	16.38
p15	H	4'-OMe	-8.449	2.64	0.991	0.112	-1.322	2.53	25.61
p16	H	3'-OMe	-8.631	2.59	1.089	0.159	-1.791	3.07	40.17
p17	H	2'-OMe	-8.433	2.49	1.019	0.171	-1.204	2.53	16.38
p18	H	3'-Cl, 4'-Cl	-8.839	3.84	0.932	0.12	-1.339	2.55	16.38
p19	H	3'-Cl, 5'-Cl	-8.870	3.98	0.865	0.194	-1.249	5.43	40.17
p20	H	3'-Me, 4'-Me	-8.526	3.74	1.244	0.045	-1.444	2.53	25.61
p21	H	3'-Me, 5'-Me	-8.600	3.74	1.260	0.081	-1.326	4.58	40.17
p22	H	4'-COOMe	-8.860	2.70	1.015	0.116	-1.523	4.96	16.38
p23	H	3'-C(O)Me	-8.751	2.27	1.197	0.041	-1.205	4.63	88.64
p24	H	4'-CH2OH	-8.595	1.64	0.978	0.114	-1.344	2.92	33.45
p25	H	3'-CH2OH	-8.652	1.64	0.958	0.206	-1.337	2.95	40.17
p26	H	4'-Br	-8.789	3.73	0.954	0.286	-1.344	2.55	16.38
p27	H	4'-CN, 2'-Me	-8.880	2.61	1.136	0.299	-1.301	3.05	36.61
p28	H	4'-CN, 3'-Me	-8.870	2.61	1.263	0.257	-1.085	3.08	40.17
p29	H	4'-CN, 3'-F	-8.997	1.98	0.997	0.263	-1.018	2.98	33.45
p30	7-F	4'-CN	-9.034	2.24	1.030	0.304	-1.541	5.36	48.97
p31	7-Me	4'-CN	-8.857	2.61	1.131	0.241	-1.362	2.97	36.61
p32	6-Me	4'-CN	-8.760	2.61	1.005	0.260	-1.307	3.01	37.97
p33	H	3'-CH(OH)Me	-8.604	1.98	1.012	0.113	-1.36	3.10	40.17
p34	H	3'-C(OH)Me2	-8.560	2.33	1.255	0.186	-1.36	5.39	85.48
p35	H	3'-COOH	-8.832	2.77	1.037	0.253	-1.34	2.57	16.38
p36	H	3'-C(O)N(Me)OMe	-8.782	2.04	1.064	0.020	-1.277	2.96	37.97
p37	H	3'-C(O)Et	-8.743	2.80	1.376	-0.033	-1.45	5.00	68.41
p38	H	3'-C(O)CH2CHMe2	-8.745	3.68	1.518	-0.002	-1.521	3.93	40.17
p39	H	3'-C(O)-(3''-F)C6H4	-8.802	3.64	1.113	0.267	-1.25	2.52	25.61
p40	H	3'-CHO	-8.845	2.25	1.058	0.109	-1.247	2.59	40.17
p41	H	3'-C(=NOH)Me	-8.686	2.27	1.209	0.131	-1.281	2.94	40.17
p42	H	3'-C(=NOMe)Me	-8.667	2.80	1.280	0.024	-1.314	3.33	37.97
p43	H	3'-C(=NOEt)Me	-8.656	3.33	1.256	0.116	-1.313	2.62	25.61
p44	H	3'-C(=NO-Bu)Me	-8.642	4.02	1.451	0.147	-1.315	2.58	16.38
p45	H	4'-C(O)-c-Pr	-8.810	2.73	1.314	0.035	-1.454	5.44	33.45
p46	H	3'-C(O)-c-Pr	-8.735	2.64	1.410	-0.037	-1.005	4.28	53.31
p47	3-I	4'-CN	-9.042	3.18	1.035	0.208	-1.557	4.74	33.45
p48	3-Cl	4'-CN	-8.951	2.75	0.966	0.103	-1.361	3.82	33.45
p49	3-Cl, 6-Cl	4'-CN	-8.991	3.21	0.713	0.089	-1.315	2.97	45.92
p50	3-Br	4'-CN	-9.006	2.92	1.160	0.209	-1.252	6.54	40.17
p51	3-Br	4'-CN, 3'-Me	-8.989	3.38	1.270	0.286	-1.472	3.17	53.68
p52	3-Ph	4'-CN	-8.682	3.91	0.831	0.386	-2.101	3.09	40.17
p53	3-(3'',5''-DiMeC6H3)	4'-CN	-8.614	4.83	1.104	0.328	-2.218	3.13	40.17
p54	3-(3''-Pyridyl)	4'-CN	-8.812	2.62	0.909	0.317	-1.507	3.13	40.17
p55	3-(2''-Furyl)	4'-CN	-8.450	3.22	1.319	0.243	-1.131	2.52	16.38
p56	3-(3''-Thienyl)	4'-CN	-8.497	3.59	1.043	-0.001	-1.195	5.08	42.68
p57	3-(3''(2''CHO)Thienyl)	4'-CN	-8.787	2.41	1.048	0.055	-1.231	5.89	33.45
p58	3-(3''(2''CH2OH)-Thienyl)	4'-CN	-8.785	2.40	1.129	0.152	-1.558	3.05	40.17

**Table 2:** Observed binding affinities^a^, pK_i(obs)_, of the substituted arylbenzofurans to the cloned human H3 receptors expressed stably in C6 cells. The pK_i (pred)LGO_ values for MLR and ANN methods are the predicted affinities obtained in the leave-group-out cross validation study.

**Compound**	**pK**_i_ (obs)^a^	**pK**_i_ (pred)LGO-MLR	**pK**_i _(pred)LGO-ANN	**pKi (pred) 3D-method (MOE)**	**Compound**	**pK**_i_ (obs)^a^	**pK**_i _(pred)LGO-MLR	**pK**_i_ (pred)LGO-ANN	**pKi (pred) 3D-method (MOE)**
p1	9.347	9.48	9.433	8.579	p30	9.367	9.408	9.372	8.303
p2	9.569	8.823	8.856	8.617	p31	8.959	9.105	9.031	8.512
p3	8.495	8.665	8.607	8.875	p32	9.114	8.655	8.452	8.405
p4	8.658	8.728	8.522	8.254	p33	9.357	8.997	9.23	8.947
p5	8.201	8.442	8.235	9.472	p34	9.602	8.796	8.809	8.226
p6	8.301	8.358	8.178	8.098	p35	7.824	8.772	8.704	8.089
p7	8.276	8.168	8.104	8.068	p36	9.208	9.558	9.585	9.731
p8	8.244	8.344	8.141	8.933	p37	9.638	9.665	9.8	8.705
p9	8.409	8.381	8.445	8.662	p38	9.167	9.385	9.67	10.110
p10	8.102	8.295	8.096	7.856	p39	8.585	8.421	8.174	8.149
p11	8.569	8.466	8.315	6.955	p40	9.398	9.34	9.321	8.859
p12	8.387	8.446	8.227	8.197	p41	9.31	9.318	9.451	8.875
p13	8.119	7.91	7.843	9.285	p42	9.377	9.233	9.346	8.629
p14	7.959	7.837	7.995	8.374	p43	8.495	8.826	8.792	8.729
p15	8.041	8.307	8.292	9.022	p44	8.367	8.56	8.438	11.379
p16	8.921	8.703	8.672	7.592	p45	9.585	9.544	9.556	8.501
p17	7.824	8.456	8.438	9.369	p46	9.678	9.679	9.49	9.326
p18	8.444	8.483	8.164	8.660	p47	9.292	9.089	8.651	9.812
p19	8.301	8.262	8.223	8.665	p48	9.409	9.152	9.238	9.324
p20	8.229	8.516	8.551	8.834	p49	9.081	8.611	9.008	9.893
p21	8.444	8.596	8.521	8.474	p50	9.538	9.278	9.108	9.699
p22	8.721	9.143	8.964	8.836	p51	8.886	9.071	9.186	9.510
p23	10.076	9.467	9.456	9.098	p52	7.678	7.42	7.715	7.684
p24	9.056	9.096	9.407	8.955	p53	7.222	7.268	7.847	7.369
p25	9.31	8.949	8.787	9.720	p54	7.886	8.568	8.453	7.848
p26	8.114	8.111	8.114	9.184	p55	8.553	8.077	8.071	8.116
p27	8.699	9.015	9.069	9.000	p56	8.678	8.228	8.054	9.264
p28	9.553	9.181	9.298	7.825	p57	9.137	9.312	9.356	8.284
p29	9.194	9.398	9.392	9.395	p58	8.721	9.246	9.219	10.571

a. Data taken from Gfesser *et al*. (22).

**Table 3:** Observed binding affinities^a^, pK_i(obs)_, of the substituted arylbenzofurans to the rat cortical H3 receptors. The pK_i (pred)LGO_ values for MLR and ANN methods are the predicted affinities obtained in the leave-group-out cross validation study.

**Compound**	**pK**_i_ ** (obs)** ^a^	**pK**_i_ ** (pred)LGO- MLR**	**pK**_i _ **(pred)LGO-ANN**	**pKi (pred) 3D-method (Hyperchem) **	**Compound**	**pK**_i_ ** (obs)** ^a^	**pK**_i _ **(pred)LGO- MLR**	**pK**_i_ ** (pred)LGO-ANN**	**pKi (pred) 3D-method (Hyperchem)**
p1	8.495	8.363	8.389	7.769	p30	8.244	8.313	8.15	8.056
p2	8.602	8.392	8.534	7.489	p31	7.796	7.955	7.978	7.796
p3	7.187	7.633	7.473	7.088	p32	8.377	7.944	7.973	7.834
p4	7.77	7.947	8.083	7.282	p33	8.553	8.329	8.345	8.198
p5	7.086	7.173	7.071	6.805	p34	8.886	9.27	9.197	9.171
p6	7.319	6.845	6.849	7.368	p35	7.18	7.427	7.182	8.500
p7	7.62	7.262	7.11	7.074	p36	8.31	8.216	8.329	9.165
p8	6.824	7.065	7.059	7.400	p37	8.638	8.554	8.669	8.157
p9	7.022	6.975	6.838	8.298	p38	7.721	7.361	7.365	7.763
p10	6.796	6.882	7.047	8.673	p39	7.276	7.222	7.108	7.525
p11	7.523	7.663	7.756	7.645	p40	8.481	8.281	8.312	7.478
p12	7.481	7.384	7.216	7.200	p41	8.509	8.161	8.301	7.837
p13	6.699	7.116	7.164	7.409	p42	8.347	7.918	7.996	7.976
p14	6.886	7.034	7.035	7.469	p43	7.292	7.427	7.359	7.905
p15	7.252	7.673	7.688	6.252	p44	7.387	6.748	6.826	10.357
p16	7.638	7.607	7.507	7.815	p45	8.194	7.82	8.013	6.397
p17	7.119	7.684	7.284	7.942	p46	8.602	8.623	8.623	8.419
p18	6.959	6.877	6.967	6.769	p47	8.076	7.602	7.465	8.472
p19	7.62	7.676	8.107	6.130	p48	8.432	7.785	7.899	8.057
p20	7.187	7.016	7.03	7.926	p49	8.013	7.853	7.704	7.898
p21	7.721	7.623	7.896	7.365	p50	8.469	8.134	8.478	7.968
p22	7.377	7.507	7.428	7.494	p51	7.796	7.832	7.946	9.552
p23	9.357	9.467	8.911	7.915	p52	6.409	6.712	6.072	7.348
p24	8.387	8.298	8.188	6.627	p53	6.076	6.078	6.564	6.479
p25	8.553	8.445	8.337	6.318	p54	7.745	7.885	7.919	7.454
p26	6.523	6.958	6.945	7.179	p55	7.377	7.338	6.936	6.704
p27	7.509	7.979	8.023	8.268	p56	7.387	8.019	8.373	7.782
p28	8.585	8.214	8.198	7.711	p57	8.237	8.361	8.42	8.123
p29	8.032	8.488	8.528	8.781	p58	7.699	7.96	7.984	6.946

**a. Data taken from Gfesser** ***et al*** **. (** 22 **).**

*MLR model*

The procedure for MLR method was performed using SPSS (ver 11.5) program as described previously (23). Briefly, the reduced data set was subjected to stepwise regression analysis to further select a limited number of descriptors significantly contributing to the prediction of binding affinities of H3 antagonists.

*ANN model*

A sigmoidal transfer function and descent gradient with momentum and adaptive learning rate back propagation was designed to predict the biological activities of H3 antagonists used in this study. The back-propagation learning algorithm is the most widely used training algorithm in multi-layered feed forward networks (17). All ANN calculations were carried out using MATLAB software with ANN toolbox for windows running on a Pentium 4 personal computer.

Before training process, the input and output values were normalized between 0.1 and 0.9. After simulation, the values of predicted data were transformed to the true values. The inputs and outputs for the ANN simulation were the values of the molecular descriptors selected by the MLR method and the *pK*_i_ values, respectively. The number of neurons in hidden layer was varied ranging from 2 to 7, and the layer consisting of 5 neurons gave the optimum results. The training parameters used in this work were as follows: The training function was traingdm; learning rate = 0.1; momentum = 0.9; and the default values were accepted for the other parameters.

*Method validation*

Predictive power of the QSAR methods was assessed by leave group out cross validation technique and the q^2^ values were calculated using the following equation:


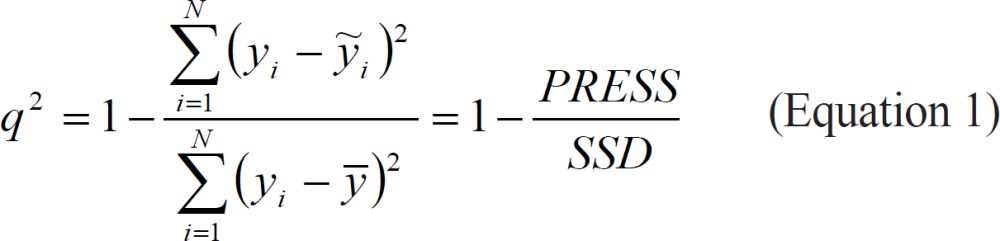


Here *SSD* is the sum of squared deviations for each actual activity value *y*_i_ (*pK*_i(obs)_) from the average activity y, over the entire data set. *PRESS*, the predictive sum of squares, is the sum of the squared differences between the actual activity *y*_i_ and the predicted activity *ỹ*_i_ (*pK*_i(pred)_).

Absolute percentage error *(APE)* of predictionwas calculated for each data point and averaged using the equations 2 and 3, respectively.


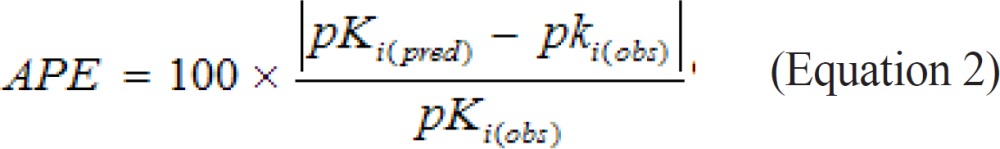



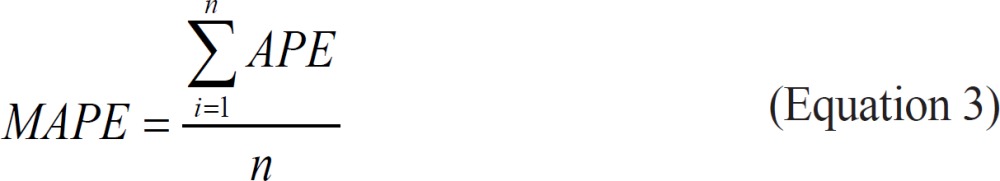


Here, *pK*_i(pred)_ and *pK*_i(obs)_ are predicted and observed binding affinities and *n* denotes the number of compounds. *MAPE* is the mean of *APE* values. Moreover, standard deviation of error of prediction *(SDEP)* was calculated to assess the distribution of error levels for rat and human data using the following equation:


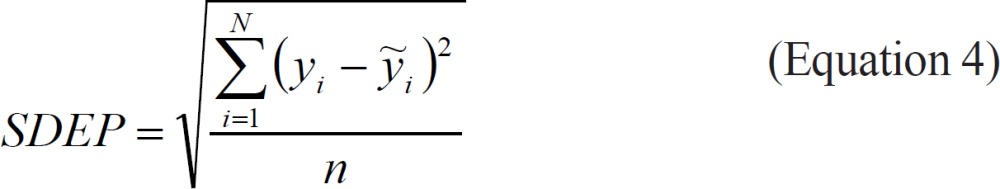


*3D-QSAR study*

Histamine H3 antagonists were superimposed using following means. (*i*) Energy minimized molecules were superimposed using three atoms from arylbenzofuran substructure common to all molecules by Overlay option of Hyperchem program. (*ii*) Using MOE program (2007.09), one of the molecules was opened and then the second molecule was superimposed using all options set to default. In the subsequent stage, the previously opened and superimposed molecules were freezed and the third molecule was loaded and superimposed onto them. The process of freezing superimposed molecules and loading and superimposing a new molecules onto the previously opened molecules was continued until all molecules were superimposed. (*iii*) In a different strategy to method *i* and *ii*, we aimed to guide superpositioning of the ligands by taking into account their relative conformations after docking them into the binding site of the histamine H3 receptor molecular model developed elsewhere (23). Flexible docking of all compounds under the investigation was carried out using GOLD program (version 2.0) running on Windows XP. Then the HASL method (version 3.30) was used for the purpose of generating 3D-QSAR model using the ligands aligned according to the procedures outlined above (20, 21).The ligands randomly divided into the training and test compounds. The training set was used to generate a 3D-QSAR model in order to predict the biological activity of the test set compounds.

## Results

Table 1 shows the chemical structures of 58 arylbenzofuran derivatives with H3 receptor antagonist activities used in this study. The table also contains the values for several molecular descriptors calculated for the structures. These descriptors were selected during the different steps of data reduction procedure using GA coupled PLS and MLR methods as outlined in Experimental section. The aim was to use not more than four descriptors in the models. The selected descriptors are the energy of highest occupied molecular orbital (*E*_HOMO_), apparent distribution coefficient at pH 7.4 (Log*D*_pH=7.4_) and two different 3D-MoRSE descriptors (*Mor*_19__V_ and *Mor*_30M_) for human data set and Log*D*_pH=7.4_, 3D-MoRSE descriptor (*Mor*_18U_), *MAXDP* topological descriptor and fragment-based polar surface area (*PSA*) for the rat data set. Equations 5 and 6 describe the ligand binding affinities to human and rat H3 receptors respectively based on the four selected molecular parameters for each correlation.

Here, n (number of data), r^2 ^(squared correlation coefficient), F (f-value) and SE (standard error) are model statistics. The significance of these molecular descriptors in describing the observed binding affinities was discussed elsewhere (23).

To process the nonlinear relationships existed between the activity and the descriptors, the ANN modeling method was employed. It was generated by using the descriptors appearing in the MLR models as inputs. A 4-5-1 neural network was developed with the optimum momentum and learning rate of 0.9 and 0.1, respectively.

A leave-group-out (LGO) cross validation technique was performed to evaluate the predictive power of the MLR- and ANN-based QSAR methods used in this study.

The observed H3 receptor binding affinities of the ligands, *pK*_i(obs)_, as well as their predicted activities using the leave-group-out cross validation method, *pK*_i(pred)_, are listed in Tables 2 and 3 for human and rat data respectively. The q^2^_LGO_ values obtained for MLR method of prediction are 0.70 and 0.79 for human and rat datasets, respectively (Table 4). Using the ANN method for prediction of the binding affinities, the q^2^_LGO_ values are 0.65 and 0.77 for human and rat datasets, respectively. The MAPE and SDEP values shown in Table 4 were also used to compare the predictive capabilities of the MLR and ANN methods.


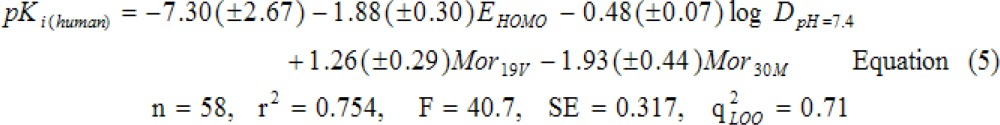



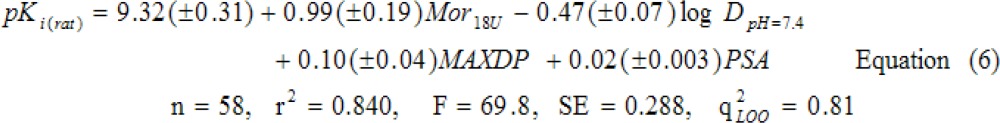


**Figure 1:**
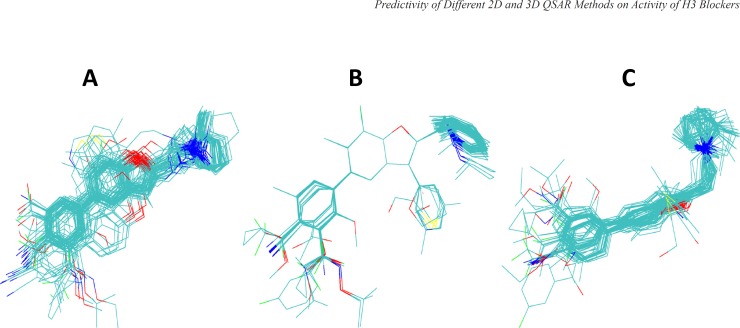
Alignments of arylbenzofuran derivatives generated by three different superpositioning approaches used in this study. Panel A shows the alignments obtained by flexible docking of molecules into the binding site of the structural model of histamine H3 receptor using GOLD program. Panel B and C are the results of superpositioning using HyperChem and MOE programs (see the text for further details).

Results from different superimposition methods on the studied arylbenzofuran H3 antagonists are depicted in Figure 1. The aligned molecules were divided into training and test sets and then the 3D-QSAR model was developed using HASL method based on the training set compounds. The activity of the test compounds were predicted using the obtained 3D-QSAR models (Tables 2 and 3) and then the absolute percentage errors of predictions were calculated (Table 4). Few rounds of model development were performed and in each round the composition of the compounds in the training and test sets were changed so that all of the compounds were given chance to be used in the test set. The results indicate that the 3D-QSAR approaches used in this study were not successful in significantly predicting the biological activity of test set compounds.

Histamine H3 receptors are autoreceptors that negatively regulate the release of histamine and other neurotransmitters such as norepinephrine, dopamine, and acetylcholine in the CNS and are believed to play a variety of physiological roles, including regulation of feeding, arousal, cognition, pain, and endocrine systems (29-31). Using the histamine H3 receptor antagonist clobenpropit, a neuroprotective role for histamine H3 receptor was also reported due to increased GABA release (32). Since the discovery of histamine H3 receptor in 1983 and cloning of its cDNA in 1999, this histamine receptor has gained the interest of many pharmaceutical companies as a potential drug target for the treatment of various important disorders, including obesity, attention-deficit hyperactivity disorder, Alzheimer’s disease, schizophrenia, as well as for myocardial ischemia, migraine and inflammatory diseases (33). Consequently, many synthetic works were conducted leading to the preclinical development of structurally diverse H3 receptor antagonists as the potential treatment tools for the above mentioned disorders (8, 11, 34-38). However, the status of drug development based on histamine H3 receptor antagonists is far behind relative to that for the H1 and H2 receptors antagonists as successful blockbuster rugs for treating allergic conditions and gastric ulcers, respectively (39).

**Table 4:** Comparison of the mean absolute percentage error (*MAPE*), standard deviation of error of prediction (*SDEP*) and q^2^_LGO_values calculated for the predictions of the binding affinities of arylbenzofuran derivatives to the human and rat H3 receptors by MLR and ANN methods.

**Statistical index**	**Human dataset **			**Rat dataset**		
	**MLR**	**ANN**	**3D-Method (MOE)**	**MLR**	**ANN**	**3D-Method (Hyperchem)**
*MAPE*	2.88	3.19	7.52	3.325	3.554	9.13
*SDEP*	0.331	0.359	0.86	0.311	0.92	0.92
q^2^_LGO_	0.7	0.65	-0.97	0.79	0.77	-0.79

The prediction of the biological activities of drug candidates is the main focus of many computer-aided drug discovery techniques. The pioneering works of generating quantitative structure-activity relationships were introduced by Hansch and coworkers in the form of MLR models. Since then many different QSAR methods were developed and used successfully in drug design and development. However, the MLR-based methods still remain one of the useful computational techniques in drug development. Here we report the QSAR studies on a set of arylbenzofuran H3 receptor antagonists using both 2D (*i.e*., MLR and ANN) and 3D (*i.e*., HASL) QSAR methods.

The purpose of QSAR studies is to select the biologically important structural descriptors and then identify the existing relations. We first used GA-PLS to reduce the number of structural features to a level manageable by MLR method. Then the MLR was used in the final feature selection step. The numbers of descriptors were kept to minimum of four in order to prevent over correlations (less than 1 descriptor per 10 compounds was selected). Equations 5 and 6 represent the MLR models generated using the four most relevant descriptors for human and rat datasets. Taking into account that the experimental procedures of obtaining the receptor affinities (*pKi*) for human and rat datasets are not the same and the H3 receptors for human and rat are not totally identical, the MLR models presented in equations 5 and 6 are reasonably similar. In our previous study we demonstrated the validity of the selected descriptors in modeling the H3 antagonist activities of the used compounds and the results were in agreement with the results of molecular modeling/ligand docking studies (23). The *E*_HOMO_ in equation 5 may indicate presence of charge transfer interaction between the benzofuran attached phenyl group of the ligands and an aromatic residues from the receptor. In equation 6, the positive model constant for *MAXDP* is indicative of a positive relationship between electrophilicity of the polar moieties of the molecule and the binding affinities to the receptor, which could be related to the charge transfer capability of the molecule and be considered as a descriptor equivalent to *E*_HOMO_ in equation 5. In both equations 5 and 6 the relative hydrophobicity of the compounds (Log *D*_pH=7.4_) is inversely related to the binding affinity. Different 3D-MoRSE descriptors, namely *Mor*_19__V_ , *Mor*_30M_ and *Mor*_18U_, were included in MLR equations 5 and 6. These descriptors are related to the 3D structures of the molecules and based on the weighting used in their calculations they are related to the volume or mass of molecules. It seems that the bigger the substituents of the molecule the higher the affinity to the H3 receptors. ANN analyses were also performed using the same set of descriptors as in the MLR method. The predictivities of MLR and ANN methods were compared using leave-group-out cross validation technique. The calculated cross-validation q^2^_LGO_coefficients as well as the *MAPE* and *SDEP* values for both MLR and ANN analyses are comparable as shown in Table 4. The statistical treatment of the results shows that there is no significant difference between the MAPE values obtained for human dataset using MLR and ANN methods (p-value of 0.22 for the paired two-tailed t-test for the means). The same is also true for the rat dataset (p-value 0.43). There are also no statistically significant differences between the variances of the errors of the predictions obtained by MLR and ANN methods for either human or rat datasets. From the numerically small values of SDEP it can be inferred that the errors are small and their distribution is not scattered.

In order to perform 3D-QSAR analysis using HASL algorithm, first the ligands were aligned using three different approaches, as mentioned in Materials and Methods. Briefly, in the first approach, Hyperchem were applied to align energy minimized molecules by superimposing three atoms selected from arylbenzofuran moiety common to all compounds. In this method molecules were kept rigid. In the second approach, MOE program was used for flexible alignment of ligands based on all available similarity terms, such as, hydrogen bond donor and acceptor, aromaticity, hydrophobicity, and partial charges. Thirdly, we used docking approach to deduce relative conformational and geometrical position of different ligands while bound to their binding site on the model built for H3 receptor in the previous study (23). The aligned ligands and their corresponding activity values were fed into HASL program to generate QSAR model. The predictive power of the 3D-QSAR model developed using the test set compounds was very poor. The calculated *MAPE* and *SDEP* values for the test compounds of human data set were 9.39 and 1.00, respectively and for rat data set these values calculated to be 10.50 and 0.96, respectively. Low level of predictive power of 3D-QSAR analyses can be related to the shortcomings of the 3D-QSAR based on the theoretical structure that we have used for the docking-guided alignment procedure in the current study in the absence of experimentally derived structure for hH3 receptor. However, other alignment protocols explained above also did not lead to the satisfactory results. Thus, one might relate the lack of predictivity seen in the current 3D-QSAR study to the method which has been used for the construction of 3D models (*i.e*., HASL). Reinvestigation of the 3D analyses using other methodologies such as CoMFA, may reveal more useful information.

In summary, the results of the current study demonstrate that the both MLR and ANN methods perform equally well in predicting the receptor binding affinities of the arylbenzofuran derived histamine H3 receptor antagonists. Although by just considering the numerical values of q^2^_LGO_ , MAPE and SDEP it seems that MLR performs marginally well, however, this is not statistically appreciable. Both of these 2D-QSAR methods were superior to HASL, a 3D-QSAR method, in predicting the activity of the arylbenzofuran H3 antagonists. The results presented in the current comparative study indicate that the application of more sophisticated and advance methods in QSAR studies does not guarantee the best predictive outcome. In many cases, like the one presented in this work, much simpler and vastly available techniques such as MLR, can predict the property of interest (*e.g*., biological activity) equally well or even better than advance methods, such as ANN and 3D based approaches.
